# Concurrent Acute Fracture and Dysplasia‐Related Fragmentation of the Medial Coronoid Process of the Ulna in a Dog—A Case Report

**DOI:** 10.1155/crve/9302458

**Published:** 2026-09-10

**Authors:** Natalie Arruda Bergamaschi, Leslie Schwarz, Mikaela Gondolfe, Fabio Brum Rosa

**Affiliations:** ^1^ Cummings School of Veterinary Medicine, Tufts University, North Grafton, Massachusetts, USA, tufts.edu; ^2^ BluePearl Pet Hospital, Charlestown, Massachusetts, USA

**Keywords:** canine elbow dysplasia, case report, fragmented medial coronoid process of the ulna, traumatic MPC

## Abstract

A 4‐year‐old neutered male Labrador retriever presented with acute‐onset nonweight‐bearing lameness of the right forelimb following play activity. Physical examination revealed crepitus and pain on elbow flexion. Radiographic examination showed a linear lucency at the cranial margin of the medial coronoid process of the ulna (MCP), with sclerosis and periosteal new bone formation, consistent with degenerative joint disease secondary to elbow dysplasia (ED). Computed tomography (CT) identified two distinct fragments adjacent to the MCP. Arthroscopic partial coronoidectomy was performed with successful retrieval of both fragments. On histopathological examination, one fragment demonstrated chronic changes, consistent with dysplasia‐related FMCP, whereas the other exhibited features consistent with acute trauma/fracture. The patient recovered well at 6‐week postoperative evaluation. This is the first reported case of concurrent developmental FMCP and traumatic MCP fracture in the same limb in a canine patient, confirmed via multimodal imaging and histopathology. This report highlights the diagnostic role of CT imaging and histopathology in differentiating overlapping pathologies and raises the question whether underlying ED plays a role in secondary traumatic injuries in dogs.

## 1. Introduction

Elbow dysplasia (ED) represents one of the most prevalent causes of forelimb lameness in dogs [1], and fragmented medial coronoid process of the ulna (FMCP) is a frequent manifestation of this condition [2, 3], with a prevalence of up to 30% [4–6]. The pathophysiology of FMCP secondary to ED is well‐established and typically results from developmental incongruity of the elbow joint, leading to abnormal stress distribution and subsequent fragmentation of the medial coronoid process of the ulna (MCP) [7–9].

In contrast, traumatic fracture of the MCP in dogs without underlying ED has been scarcely reported [7, 10] and follows a different pathophysiological mechanism. Previous reports have attributed this injury to compressive forces and rotational stress applied to the MCP against the radial head during biceps brachii muscle contraction [8]. This mechanism suggests an acute traumatic event rather than the chronic developmental process characteristic of FMCP in dysplastic elbows. Although conventional radiography remains the primary modality for the initial assessment of ED in dogs, the inherent complexity of this developmental skeletal disorder and the frequent superimposition of articular structures may necessitate multimodal diagnostic approaches. Normal radiographic findings do not exclude the presence of medial coronoid disease [11] and are known to underestimate the extent of underlying osseous pathology [12]. Notably, Tan et al. [9] reported that all 24 dogs presenting with acute MCP fractures demonstrated unremarkable radiographic findings, further underscoring the limitations of radiography as a sole diagnostic tool. Computed tomography (CT) has become increasingly accessible in veterinary medicine and has demonstrated superiority over conventional radiography in the evaluation of joint disease and comprehensive assessment of the dysplastic elbow [13–15].

Although acute MCP fractures are frequently managed surgically via arthroscopic removal of the fragment [16, 17], the optimal treatment strategy for FMCP associated with ED remains controversial, and surgical intervention is not universally indicated [18]. A precise diagnosis is therefore critical, as the distinction between an acute traumatic fracture and FMCP secondary to ED directly dictates the treatment pathway and potential prognosis.

This case report describes a dog diagnosed with concurrent FMCP due to ED and acute, traumatic fracture of the MCP on the same limb. Following diagnosis through conventional radiography and CT, the patient underwent arthroscopic removal of both coronoid pieces and recovered uneventfully. For the purposes of this report, both medial coronoid pieces are collectively referred to as “fragments” prior to histopathological evaluation. In this case report, we aimed at describing the imaging, surgical, and histopathological findings of this uncommon dual pathology, and highlight the importance of advanced imaging and histopathology in the evaluation of elbow disease in dogs.

## 2. Case Description

A 4‐year‐old 33 kg, neutered male Labrador retriever was presented to the emergency room for evaluation of acute onset lameness of the right thoracic limb, after playing with another dog the day prior. The patient had no previous pertinent medical conditions, and its familial history was unknown.

**Figure 1:**
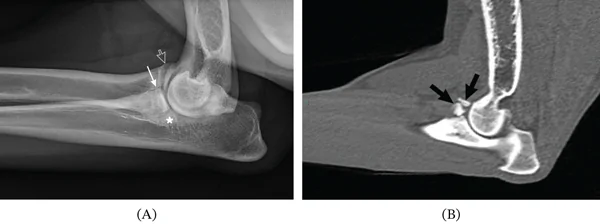
(A) Lateral radiograph of the right elbow shows a thin linear lucency at the MCP (white arrow), suspected to be a fracture or identification of the site of an FMCP due to tangential X‐ray beam aligning with the fragmentation site. Associated sclerosis ( ^∗^) and mild periosteal new bone formation along the margins of the elbow joint (hollow arrow) are also present. (B) Reconstructed sagittal CT image of the same elbow depicting two isolated fragments (black arrows) cranial to the MCP.

**Figure 2:**
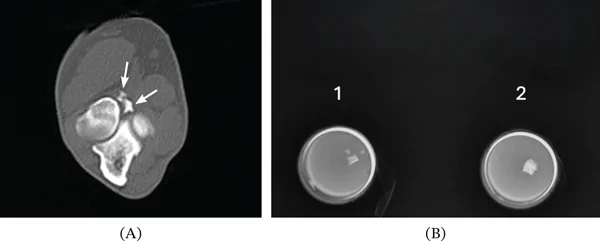
(A) Reconstructed transverse CT image of the right elbow showing two separate fragments associated with the MCP (arrows). (B) Radiograph of the surgically removed coronoid pieces; Fragment 1 (Marker 1) was suspected to be acute, and Fragment 2 (Marker 2) was suspected to be chronic.

On initial physical examination, the patient showed intermittent, nonweight‐bearing lameness of the right thoracic limb, with crepitus and pain upon flexion of the right elbow. On two‐view radiographs (80 kVp, 3.2 mAs; small focus Del Medical Canon; manufactured in July 2016, North Charleston, South Carolina) of the right elbow (Figure 1A), there was a thin linear lucency at the cranial margin of the MCP with associated sclerosis and mild periosteal new bone formation along the margins of the elbow joint. These latter findings were consistent with degenerative joint disease, likely secondary to ED. The linear lucent region at the MCP was suspected to be a fracture or identification of the site of an FMCP because of a tangential X‐ray beam aligning with the fragmentation site. A CT study was pursued for further evaluation of the elbows (Figures 1B and 2A) prior to arthroscopy. On CT, two isolated fragments were identified adjacent to the MCP of the right elbow. The largest, at the base of the MCP, was well mineralized and had a thin, well‐demarcated transverse line separating it from the intact portion of the MCP. The smaller and less mineralized fragment, at the tip of the MCP, had less well‐defined margins. The left elbow showed no abnormalities on CT.

Surgical intervention was performed via elbow arthroscopy and included partial coronoidectomy, MCP debridement, and retrieval of both coronoid pieces. The fragments were assessed at the time of arthroscopy by the surgeon; Fragment 1 (Marker 1) was thought to be acute and Fragment 2 (Marker 2) thought to be chronic. Following arthroscopic removal, the fragments were radiographed (Figure 2B) and identified as Fragments 1 (Marker 1) and 2 (Marker 2).

**Figure 3:**
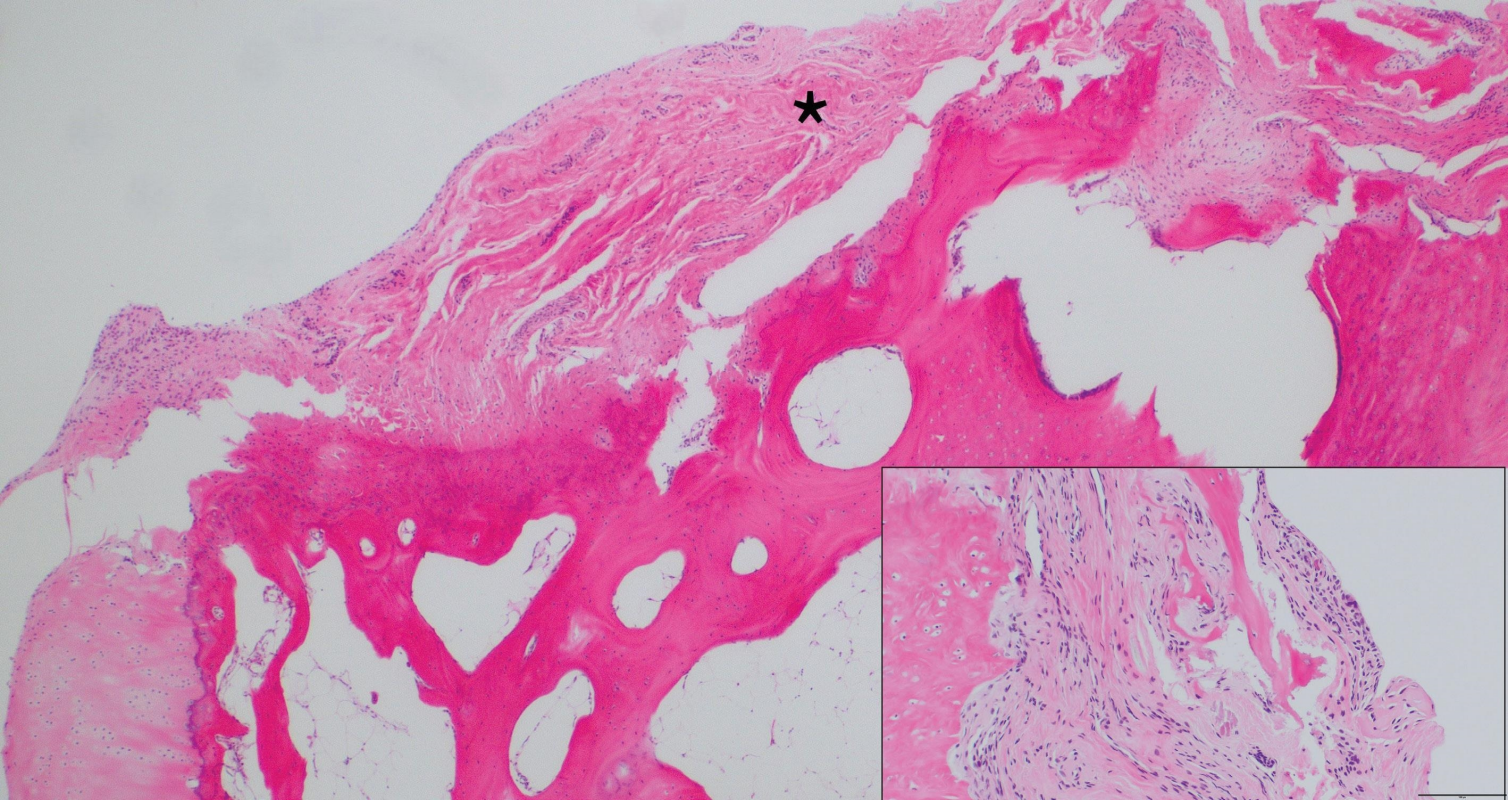
Histopathological findings (H&E stain) of Fragment 2 (chronic). The fibrous periosteum is mildly thickened by fibrous connective tissue ( ^∗^). Higher magnification shows perivascular lymphocytes and plasma cells, along with small microfractures and mild bone resorption.

**Figure 4:**
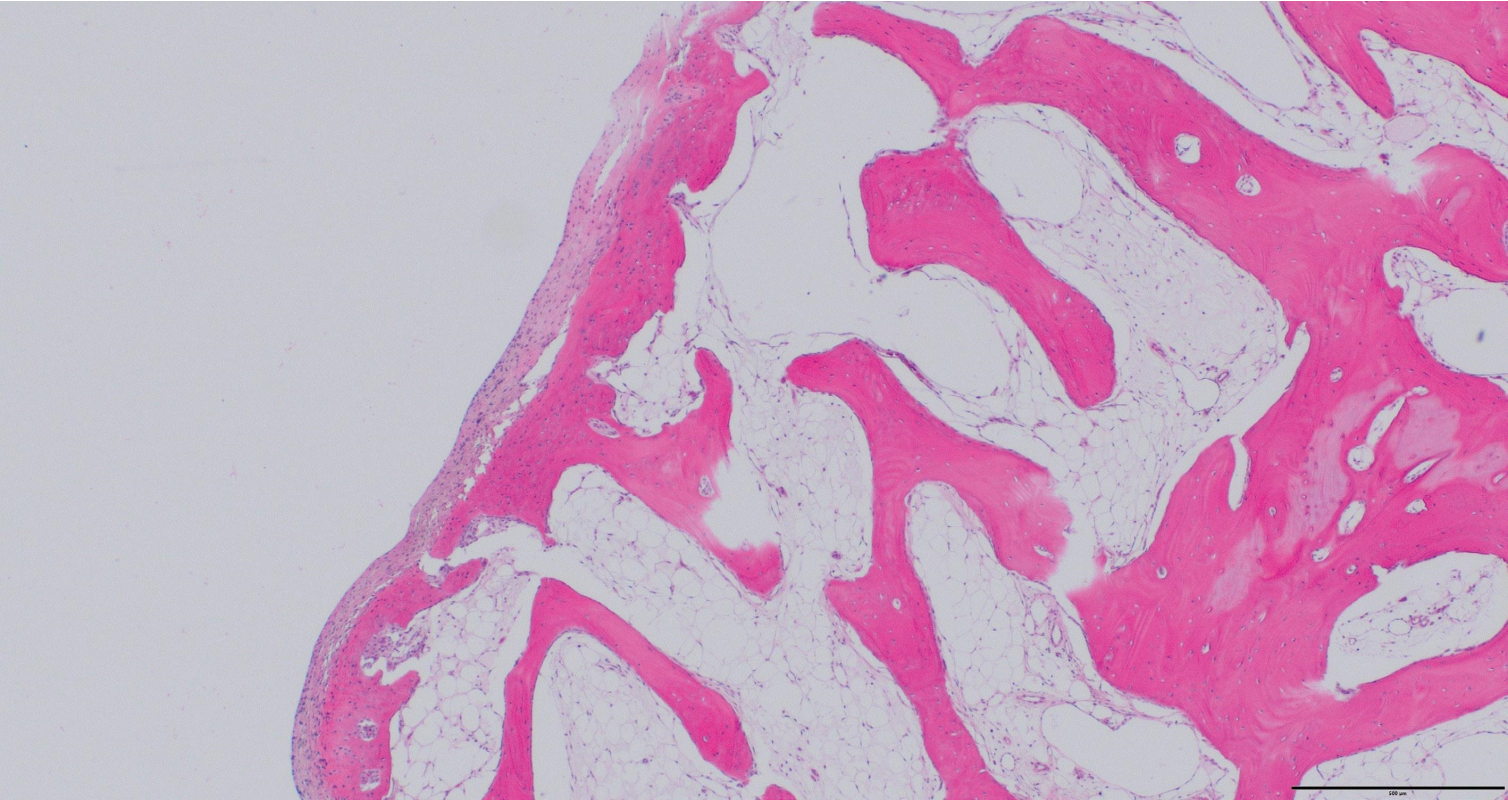
Histopathological findings (H&E stain) of Fragment 1 (acute). The fibrous periosteum is within normal limits and is markedly thinner than Fragment 2.

Histopathology revealed that Fragment 2 had slightly thicker articular cartilage, which was thought to be an anatomical variation. It also showed a mildly thickened fibrous periosteum with rare perivascular lymphocytes and plasma cells, along with small microfractures and mild bone resorption. Based on this latter feature, Fragment 2 exhibited more signs of chronicity than Fragment 1 (Figures 3 and 4).

When assessed together, the imaging (radiographs and CT), surgical, and histopathological findings were in agreement that the larger base fragment, seen radiographically as the lucent linear structure, was the acute traumatic fracture of the MCP, whereas the smaller more superficial medial coronoid structure represented an FMCP.

The patient had an uneventful recovery postarthroscopy with no lameness at the 6‐week recheck postsurgery. At follow‐up, the owner reported no lameness 21 months after the procedure.

## 3. Discussion

This case presents imaging features and surgical management of an unusual orthopedic pathology involving concurrent FMCP secondary to ED and subsequent traumatic fracture of the MCP in the same joint in a canine patient. To the authors′ knowledge, this dual pathology has not been previously reported in the veterinary literature, representing a novel clinical, imaging, and pathological observation.

The altered biomechanics from ED leading to FMCP are well described [5, 6] and involve elbow joint incongruity leading to uneven force distribution, causing excessive load on the MCP [5]. Hypothetically, ED could create conditions that increase susceptibility to acute traumatic MCP fracture during routine activities or minor, repetitive trauma. This type of fracture mechanism is analogous to insufficiency fractures in humans [19–21], in which bones with compromised mechanical integrity fail under normal physiological loads. More specifically, the subchondral bone underlying the dysplastic elbow undergoes microdamage and remodeling over time [5]. Consequently, forces that would ordinarily be tolerated during routine weight‐bearing activities may become sufficient to propagate an acute fracture through a now structurally weakened region. The additive effect of routine forces on a remodeled bone region may explain how this fracture occurred during a period of apparently unremarkable activity in the patient presented here. It remains uncertain, however, whether a causal relationship between ED and secondary acute traumatic MCP injury exists. If aberrant elbow biomechanics were a meaningful predisposing factor, a higher prevalence of this dual pathology would be anticipated; to date, this has not been established. Notably, however, bone fragments retrieved during arthroscopy are anecdotally infrequently submitted for histopathological analysis, suggesting that the true prevalence of this dual pathology may be underestimated.

This case conforms to several features of the established demographic profile for medial compartment elbow disease. Male dogs are twice as likely to develop ED [11], and Labrador retrievers are among the overrepresented breeds [6, 14]. Additionally, although ED commonly affects both elbow joints, unilateral disease, such as in our patient, is well recognized, with reported prevalence up to 51% in dogs with elbow lameness [14, 15]. Conversely, the most common age of diagnosis for FMCP secondary to ED is younger than 1 year—most commonly between 4 and 7 months [5]—and typically less than 18 months [16], reflecting the developmental nature of the condition. The patient in this report was older than this typical window of presentation; however, this does not necessarily preclude an ED‐related etiology, as up to 25% of dogs with ED may exhibit subclinical or subtly compensated lameness [5]. Additionally, mild or slowly progressive lameness may escape owner detection until a precipitating event produces a marked and sudden deterioration in limb function. It is therefore plausible that underlying lameness‐related ED was present in this patient from a younger age but went unrecognized, with clinical presentation delayed until the acute fracture generated a level of pain and dysfunction that gained the owner′s attention.

The histopathological differences observed between the two medial coronoid pieces in this case are consistent with previously published findings for fragmentation from traumatic fractures and FMCP associated with ED [10, 12] and provided the objective evidence distinguishing the two processes occurring concurrently in the same limb. Establishing the nature of the pathological process responsible for MCP fragmentation carries direct clinical consequences. Surgical removal of a dysplastic fragment alone is not universally recommended [1–4], given that the underlying joint incongruity persists and degenerative joint disease will likely ensue regardless. However, the additional presence of an acute traumatic fragment may alter this calculus and strengthen the indication for a surgical approach. This represents a clinically important scenario for which no established guidelines currently exist. The described case report highlights the need for CT evaluation and histopathological characterization to inform individualized treatment options.

Several limitations of this report must be acknowledged. Although a nearly 2‐year follow‐up period on our patient was obtained, it is well recognized that ED is associated with the progressive development of osteoarthritis, ongoing cartilage degradation, and potential recurrence of lameness several years following surgical intervention [2, 4, 17]. Whether the coexistence of a superimposed, acute traumatic fracture modifies the long‐term disease course—either by accelerating joint degeneration through the additional cartilage insult at the time of trauma, or by offering a more favorable prognosis if the acute fracture fragment is identified and removed early before significant chondral damage occurs—remains unknown. Extended follow‐up, potentially with use of force plate analysis [22], would be required to draw meaningful conclusions regarding the long‐term prognosis specific to this dual presentation. Additionally, as a single case report, generalization of these findings is inherently limited, and studies with larger cohorts would be necessary to establish the true prevalence of concurrent FMCP and traumatic MCP fracture in dogs with ED.

In conclusion, the diagnostic complexity of this case emphasizes the need for advanced imaging and histopathology protocols that can reliably differentiate between overlapping pathological processes. Traditional radiographic assessment may prove insufficient when developmental and traumatic MCP pathologies coexist. In the case of dysplastic elbows, morphological changes can obscure acute fracture lines or mimic traumatic fragmentation, yielding different treatment recommendations. Multimodal imaging approaches, incorporating high‐resolution CT with multiplanar reconstructions, and histopathology may be essential for accurate characterization of dual pathology.

## Funding

No funding was received for this manuscript.

## Consent

Written informed consent for publication of clinical details and/or clinical images was obtained from the patient′s owner.

## Conflicts of Interest

The authors declare no conflicts of interest.

## Data Availability

The data that support the findings of this study are available from the corresponding author upon reasonable request.
